# Acceptability and feasibility of enhanced cognitive behavioral therapy (eCBT) for children and adolescents with obsessive–compulsive disorder

**DOI:** 10.1186/s13034-021-00400-7

**Published:** 2021-09-04

**Authors:** Lucía Babiano-Espinosa, Lidewij H. Wolters, Bernhard Weidle, Scott N. Compton, Stian Lydersen, Norbert Skokauskas

**Affiliations:** 1 Regional Centre for Child and Youth Mental Health and Child Welfare, Department of Mental Health, Norwegian University of Science and Technology, Trondheim, Norway; 2 Department of Psychiatry and Behavioral Sciences, Duke University School of Medicine, Durham, NC USA; 3 Department of Child and Adolescent Psychiatry, St. Olav’s University Hospital, Trondheim, Norway

**Keywords:** Obsessive–compulsive disorder (OCD), Cognitive behavioral therapy (CBT), Enhanced cognitive behavioral therapy (eCBT), Children, Adolescents

## Abstract

**Introduction:**

Obsessive–compulsive disorder (OCD) is a disabling mental health disorder affecting 1–3% of children and adolescents. Cognitive behavioral therapy (CBT) is recommended as the first-line treatment, but is limited by accessibility, availability, and, in some cases, response to treatment. Enhancement with Internet technologies may mitigate these challenges.

**Methods:**

We developed an enhanced CBT (eCBT) treatment package for children and adolescents with OCD to improve treatment effect as well as user-friendliness. This study aims to explore the feasibility, acceptability, and preliminary effectiveness of the eCBT intervention. The eCBT protocol consists of 10 face-to-face and 12 webcam sessions delivered in 14 weeks. CBT is enhanced by a smartphone application (app) for children and parents to support and monitor treatment, psychoeducative videos, and therapist-guided webcam exposure exercises conducted at home. Assessments were performed at baseline, post-treatment, and at 3- and 6-month follow-up. Primary measures of outcomes were the the Client Satisfaction Questionnaire-8 (CSQ-8) (acceptability), treatment drop-out (feasibility) and the Children’s Yale-Brown Obsessive–Compulsive Scale (CY-BOCS) (preliminary effectiveness).

**Results:**

This paper describes 25 patients with OCD (aged 8–17 years) treated with eCBT. Results indicated that children and parents were satisfied with eCBT, with CSQ-8 mean scores of 27.58 (SD 0.67) and 29.5 (SD 3.74), respectively (range 8–32). No patients dropped out from treatment. We found a mean of 63.8% symptom reduction on the CY-BOCS from baseline to post-treatment. CY-BOCS scores further decreased during 3-month and 6-month follow-up.

**Conclusion:**

In this explorative study, eCBT for pediatric OCD was a feasible, acceptable intervention demonstrating positive treatment outcomes.

## Introduction

Obsessive–compulsive disorder (OCD) is a disabling mental health disorder affecting 1–3% of children and adolescents [1, 2], leading to significant impairment [3] and reduced quality of life [4]. Without treatment, OCD has a chronic course in 40–60% of cases [5, 6]. Cognitive behavioral therapy (CBT) is the first line of treatment for children with OCD [7–9]. However, from a global perspective, not all patients who need CBT receive it, due to its limited availability (i.e., lack of experienced therapists, geographic barriers, high costs) [10–12].

The use of Internet technology may address some of these challenges. Several researchers have attempted to improve the availability and accessibility of CBT by developing low-cost and easily accessible OCD self-help treatment programs [13–16]. In addition, smartphone applications (apps) have been used to overcome geographic barriers and to improve compliance with Exposure and Response Prevention (ERP) [15, 17]. A recent systematic review concluded that Internet-based CBT (iCBT) programs for pediatric OCD are feasible, acceptable, and possibly effective. However, the systematic review was based on six studies (N = 96), and the authors called for more studies on iCBT for pediatric OCD before reaching firmer conclusions [18].

Exposure and Response Prevention can be affected by low motivation, avoidance behavior, and the lack of possibilities to carry out therapist-guided exposure exercises in the patient’s home. Children often do their homework best in the first days after a treatment session, but efforts then decrease gradually. In a study of internet delivered CBT with minimal therapist contact by Lenhard et al., [15] half of the sample were satisfied with the internet format most of the time but would have liked to meet with a clinician occasionally. Combining traditional face-to-face sessions with webcam sessions at home and an app is one possible way to address these problems, to increase treatment intensity, and to make use of children’s and adolescents’ fascination with internet technology.

In order to address the challenges with traditional CBT highlighted above, we developed an enhanced cognitive behavior therapy (eCBT) treatment package for children and adolescents with OCD. The main goal of this package is to improve treatment satisfaction and compliance, to intensify exposure and response prevention, and thereby improve treatment outcomes. This study reports on the first 25 patients to receive eCBT and explores acceptability and feasibility and initial treatment outcomes. Our hypotheses are that eCBT for children and adolescents with OCD will be acceptable for both patients and their parents, and feasible for users as well as treatment providers. In addition, we hypothesize that eCBT will have positive outcomes in a preliminary evaluation.

## Methods

### Design

This case series study evaluated outcomes for 25 patients treated with eCBT for OCD over a 25-month period (from January 2018 to February 2020). Assessments were performed pre- and post-treatment, and at 3- and 6-month follow-up. Efficacy measures were completed at all assessment points. Treatment acceptability was examined at the post-treatment assessment.

### Intervention

Enhanced CBT (eCBT) is an innovative treatment package for children and adolescents with OCD. It integrates modern technology with well validated principles of CBT. eCBT was developed by academic experts in the treatment of OCD, experts in IT, and media developers. Advice from service users who had received traditional CBT and clinicians about what could help them to structure and improve treatment was incorporated in the final version of eCBT. eCBT employs evidence-based principles of CBT taken specifically from Norwegian [19] and Dutch [20] treatment manuals for pediatric OCD. Similar to traditional CBT, eCBT contains psychoeducation, Exposure and Response Prevention (ERP), cognitive interventions, and relapse prevention strategies. In addition to traditional face-to-face treatment sessions, eCBT enhances treatment by offering sessions in situ (e.g., in the child’s home) via webcam, allowing more frequent therapist contact and the ability to conduct more ecologically valid exposure exercises. Parents are also actively involved in the treatment.

eCBT covers a 14-week treatment period. The first part of the treatment (weeks 1–5) consists of weekly (45-min) face-to-face sessions, equivalent to traditional CBT. At least one of the four psychoeducation videos was demonstrated by the therapist during the first sessions and patients and families are encouraged to watch all of them at home. All videos were available on the app to watch them at any time. As soon as the child starts with ERP exercises at home (week 2), treatment is supplemented by a weekly (15-min) videoconferencing meeting, resulting in two appointments with the therapist per week. Simultaneously children will start logging their ERP homework in the app.

During the webcam sessions, the therapist guides the child while carrying out an ERP exercise at home or at another location, if applicable. In the second part of the treatment, from week 6 onwards, the frequency of the face-to-face sessions is reduced from weekly to bi-weekly, while the frequency of webcam sessions (guided ERP at home) is increased, resulting in one face-to-face session and two videoconferencing meetings in a two-week period. This schedule provides the therapist with extra tools to ensure adequate execution of the ERP exercises in a natural environment. However, total therapist time for eCBT is equivalent to traditional CBT. The default frequency of sessions could be altered and personalized to patients’ needs but could not exceed the total number of sessions (a maximum of 10 face-to-face sessions and up to 12 shorter webcam sessions).

The eCBT package is fully integrated into the treatment process and consists of a smartphone app for children, a smartphone app for parents, and a web-based computer application for therapists. Each platform is interconnected. The main goals of the eCBT package are to increase treatment adherence, provide more ecologically valid exposure exercises, and encourage parents’ involvement in the treatment process. The eCBT package further contributes to personalizing treatment to individual needs. The app provides information about OCD and CBT (i.e., psychoeducation videos showing animated narratives of children with OCD), supports and structures ERP exercises at home, and closely monitors treatment progress (short and frequent assessments with direct feedback to the patient and therapist). The web-based platform for therapists serves a coordinating and monitoring function. The app can be used in the treatment sessions together with the therapist or independently at home. It was developed for the Android system only, and children who had an iPhone had to borrow an Android phone from the study.

### Participants

Participants were eligible if they met the following criteria: age 7–17 years; primary DSM-5 diagnosis of OCD; and Children’s Yale-Brown Obsessive–Compulsive Scale (CY-BOCS) score ≥ 16. Exclusion criteria were psychiatric comorbidity which had a higher treatment priority than OCD and made participation clinically inappropriate (e.g., primary anorexia nervosa, depression with suicidality, or psychosis); ongoing psychological treatment for OCD other than eCBT; significant developmental delays; or insufficient understanding of the Norwegian language. Concurrent medications were allowed during the study.

## Measures

### Acceptability measures

The Client Satisfaction Questionnaire 8 (CSQ-8) examines client satisfaction with (mental) health services. The questionnaire consists of eight items that are answered on a 4-point Likert scale. The total score ranges from 8 to 32, with higher scores indicating more satisfaction [21, 22].

The User Experience Questionnaire (UEQ) measures users’ experiences with interactive products. The UEQ contains six scales (attractiveness, perspicuity, efficiency, dependability, stimulation, and novelty) and has 26 items. Each item presents two opposites from one dimension (for example, not understandable (1) to understandable (7)) [23].

The Treatment Evaluation Questionnaire (TEQ) was developed for this study to assess the experiences of children and parents with eCBT. The TEQ consisted of 10 and 11 items (parent and child version respectively) that were answered on a 5-point Likert scale (from very helpful to not helpful at all). In addition, participants were encouraged to share suggestions and comments.

### Feasibility measures

Treatment drop-out was the primary measurement of feasibility and was defined as premature cessation of treatment before completing the planned number of sessions according to the protocol and not due to patients’ recovery. A session integrity form was developed to monitor eCBT treatment sessions and to record deviations from the eCBT treatment manual. The session integrity form was completed by the therapist after each session. Session integrity forms were inspected for deviations by two of the authors (LBE and BW).

The modified Barriers to Treatment Participation Scale (BTPS) is a self-reporting questionnaire to measure perceived barriers to participation in treatment. Barriers include practical obstacles related to participation, perceptions that treatment is (too) demanding, not helpful, or of little relevance to the child’s problems, and a poor alliance with the therapist [24]. For this study, the BTPS was modified, and items not applicable for Norwegian clinical services and this study were omitted. The modified BTPS consisted of 27 items for the parents’ version. For the children’s version, 15 items from the parents’ version, applicable to children’s situation were reworded. All items were answered on a 5-point Likert scale (1 = never a problem to 5 = very often a problem).

### Efficacy measures

The Children’s Yale-Brown Obsessive–Compulsive Scale (CY-BOCS) is a clinician-rated, semi-structured interview used to assess the severity of OCD symptoms. The CY-BOCS interview consists of 10 items measuring five dimensions (time occupied by symptoms, interference, distress, resistance, and degree of control over symptoms) of obsessions and compulsions. The CY-BOCS total score ranges from 0 to 40 (clinical cut-off = 16). CY-BOCS shows reasonable reliability and validity [25] [26].

The Clinical Global Impression (CGI) measures symptom severity, treatment response, and efficacy in treatment studies. It scales for severity and improvement. The Severity Scale (CGI-S) is a 7-point scale from 1 (normal) to 7 (among the most extremely ill patients). The Improvement Scale (CGI-I) is also a 7-point scale from 1 (very much improved) to 7 (very much worse) [27]. The CGI is included in the CY-BOCS.

### Other measures

The Schedule for Affective Disorders and Schizophrenia for School-Age Children –Present and Lifetime Version (K-SADS-PL) is a semi-structured diagnostic interview that assesses child and adolescent psychopathology according to DSM-IV criteria [28]. The K-SADS-PL was used to confirm inclusion criteria, i.e., a diagnosis of OCD and to assess comorbidities, that could influence treatment priorities. Symptoms can be classified as “not present”, “possible”, “in remissions” or “certain*”.* In this study OCD and other diagnosis were given based on “certain” symptoms only.

Demographic information, symptom development and treatment history were collected systematically from parents with a standardized questionnaire.

### Procedures

Patients were referred to the Department of Child and Adolescent Psychiatry, St. Olav’s University Hospital, Trondheim. Patients meeting the study’s eligibility criteria were informed about the eCBT study. They had a reasonable amount of time to consider participation and to ask questions. After informed consent was obtained, patients were enrolled into the study. During treatment, all patients were offered Android Mobile Phone with preinstalled eCBT for free. However, patients were allowed to use their own Android Phone, if they preferred, as eCBT was produced for Android system.

At the start of eCBT, participants received technical assistance with app activation and videoconferencing software. If they encountered technical problems during treatment, they were advised to contact the project team. Assessments were carried out at baseline, after completion of eCBT treatment, and at 3 and 6 months after treatment*.* The CBT therapists involved were either licensed clinical psychologists (n = 6) or child and adolescent psychiatrists (n = 2). All were trained in CBT with ERP and had weekly supervision by one of the co-authors (BW).

A qualified mental health professional assessed obsessive–compulsive and other psychiatric symptoms, using the K-SADS-PL prior to referral to the OCD team. An independent evaluator—a psychologist not involved in the treatment of any participant—conducted and scored the CY-BOCS interviews. However, in some few cases when he was not available, another therapist not informed about the treatment of the participant carried out this assessment.

### Descriptive statistics

For treatment acceptability the Client Satisfaction Questionnaire 8 (CSQ-8) group mean scores and standard deviations for each item were calculated for children and parents separately. For the User Experience Questionnaire (UEQ), we calculated group means and standard deviation for all six subscales for children as well as parents. The Treatment Evaluation Questionnaire (TEQ) was analyzed tallying each participant’s (children and parents) rating for each item.

Treatment feasibility was examined by tallying treatment dropouts. For the modified Barriers to Treatment Participation Scale (BTPS) the frequency of perceived barriers to treatment is descriptively summarized.

Treatment outcomes for OCD are assessed by calculating percentage improvement from baseline to post-treatment on the CY-BOCS. The criterion for treatment response was ≥ 30% symptom reduction, and the criterion for clinical remission was a CY-BOCS score ≤ 10 [29–32]. Longitudinal outcomes on the CY-BOCS were assessed at baseline, post-treatment, and at 3- and 6-month follow-up, and are presented in group mean scores and standard deviations at the respective assessment points. SPSS software, version 25, was used [33].

### Ethics

The study was approved by the Regional Committee for Medical and Health Research Ethics (No2016/716/REK Nord) and registered with the ISRCTN (https://www.isrctn.com/) registry (trial ID: ISRCTN37530113) [34]. The study procedures were in accordance with the principles of the Declaration of Helsinki [34] and Good Clinical Practice (GCP) standards [35].

## Results

Between January 2018 and February 2020, 45 eligible patients at the Departments of Child and Adolescent Psychiatry, St. Olav’s University Hospital, Trondheim, and Aalesund Hospital were informed about the present study. Twenty-six patients accepted eCBT, 11 preferred a brief intensive CBT group treatment, and 8 patients refused any treatment for OCD. The first included patient did not have webcam sessions due to initial technical problems and was therefore excluded. All 25 patients enrolled in the eCBT treatment program completed the treatment.

Fourteen patients had comorbid disorders (as confirmed with the K-SADS) including tic disorder, anxiety disorder, attention deficit hyperactivity disorder (ADHD), eating disorder, and autism spectrum disorder. Table 1 provides more details about patients’ socio-demographic and clinical characteristics.

**Table 1:** Clinical characteristics and assessments at baseline, post-treatment, and at 3- and 6-month follow-up (n = 25)

Age (years)	Gender	Comorbidities (K-SADS)	Medication (dose/day)	Number of sessions: face-to-face/webcam	CY-BOCS total score				CGI–Severity / CGI-Improvement			
					*Pre*	*Post*	*3 m*	*6 m*	*Pre*	*Post*	*3 m*	*6 m*
12	M	SAD, GAD, ADHD, Tic disorder, ASD	Guanfacine (4 mg)	14/1	34	17	0	0	6	3/2	1/1	1/1
17	F	None	None	9/6	25	6	0	0	5	1/1	1/1	1/1
9	M	Depression, Eating disorder	None	7/1	18	11	11	21	4	1/2	1/3	5/3
16	F	None	None	10/11	18	14	18	N.A	4	4/3	5/3	N.A
10	F	SAD, Spec. phobia, ADHD, Tic disorder	None	6/1	25	0	0	0	5	1/1	1/1	1/1
10	F	GAD	None	6/3	24	0	0	0	4	1/1	1/1	1/1
17	M	Tic disorder	None	10/2	35	22	20	18	6	4/3	2/4	1/4
15	F	None	None	10/2	24	2	2	2	5	1/1	1/1	1/1
13	M	Tic disorder	None	10/6	31	8	0	0	5	4/1	1/1	1/1
12	F	Eating disorder	Risperidone (1 mg)	14/1	34	33	N.A	5	6	6/3	N.A	1/1
13	F	GAD	None	10/7	31	23	N.A	N.A	6	5/3	N.A	N.A
13	F	None	Methylphenidate (40 mg)	9/4	20	5	2	1	4	1/1	1/1	1/6
14	F	None	None	12/9	27	7	4	19	5	1/1	2/1	4/3
17	M	ASD	None	3/2	21	0	N.A	N.A	5	1/1	N.A	N.A
8	F	None	None	7/5	25	0	0	0	4	1/1	1/1	1/1
13	M	None	None	10/6	30	0	0	0	4	1/1	1/1	1/1
16	M	N.A	None	7/7	25	20	N.A	N.A	5	4/3	N.A	N.A
13	F	ASD	None	10/3	29	12	1	0	6	3/1	1/2	1/1
13	F	None	None	9/4	18	12	12	12	4	3/3	3/3	2/3
8	M	None	None	8/3	31	0	0	0	5	1/1	1/1	1/1
14	M	Bipolar disorder	Aripiprazole (20 mg)	4/6	21	17	N.A	N.A	4	3/3	N.A	N.A
17	F	None	None	8/9	32	0	0	0	5	1/1	1/1	1/1
8	M	PTSD	None	7/5	20	10	2	0	4	1/2	1/1	1/1
16	F	Spec. phobia, ADHD	Methylphenidate (20 mg)	11/4	21	14	6	3	5	2/1	2/1	1/1
14	M	ASD, Tic disorder	None	9/4	25	0	0	0	3	1/1	1/1	1/1

*K-SADS* Kiddie Schedule for Affective Disorders and Schizophrenia, *CGI* Clinical Global Impression scale, *CY-BOCS* Children’s Yale-Brown Obsessive–Compulsive Scale, *GAD* Generalized Anxiety Disorder, *SAD* Separation Anxiety Disorder, *ADHD* Attention Deficit Hyperactivity Disorder, *ASD* Autism Spectrum Disorder, *PTSD* Post Traumatic Disorder, *N.A.* Not available, *M* male, *F* female

### Acceptability

The Client Satisfaction Questionnaire 8 (CSQ-8) was filled in by 22 children and 18 parents. CSQ-total scores ranged from 23 to 32 (M = 27.7, SD = 3.9) for children, and from 24 to 32 (M = 29.5, SD = 3.7) for parents (Table 2). Participants scored all items 3 “mostly satisfied” or 4 “highly satisfied”. There was no statistically significant difference between CSQ-8 scale scores for parents and children.

**Table 2:** Client Satisfaction Questionnaire (CSQ-8): Children’s and parents’ rating

Item	Children (n = 22); mean (SD)	Parents (n = 18); mean (SD)
1. Quality of service	3.41 (0.59)	3.67 (0.49)
2. Kind of service wanted	3.50 (0.60)	3.61 (0.50)
3. Needs met	3.30 (0.76)	3.56 (0.51)
4. Would recommend to friend	3.65 (0.57)	3.78 (0.43)
5. Satisfaction with help received	3.32 (0.84)	3.67 (0.49)
6. Dealt with problems	3.52 (0.60)	3.72 (0.46)
7. Overall satisfaction	3.48 (0.67)	3.83 (0.38)
8. Would return to program	3.40 (0.67)	3.67 (0.49)
Total score (range 8–32)	27.58 (0.67)	29.5 (3.74)

**Fig. 1:**
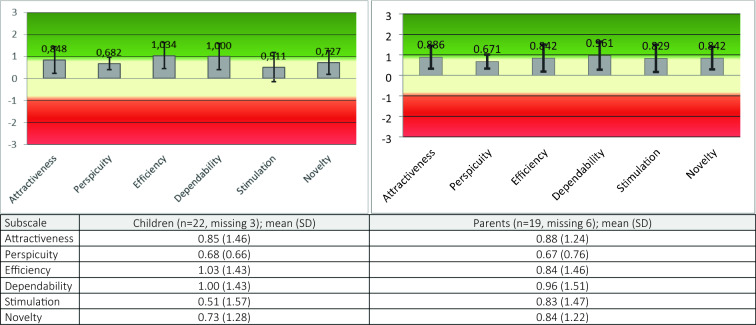
User Experience Questionnaire (UEQ): Children’s and Parents’ rating

The User Experience Questionnaire (UEQ) was filled in by 22 children and 19 parents. All subscales were rated as ‘average’ by both children and parents (Fig. 1).

**Fig. 2:**
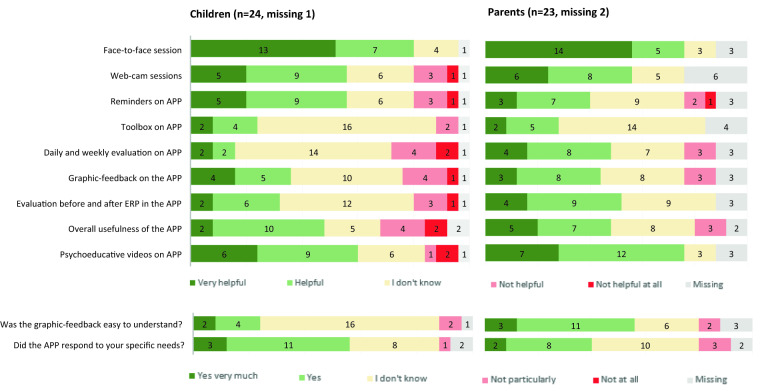
Treatment Evaluation Questionnaire

The Treatment Evaluation Questionnaire (TEQ) was completed by 24 children and 23 parents (Fig. 2). One of the parents had only filled in 2 items. Most positively evaluated were the face-to-face sessions, reported as being helpful or very helpful by 20 children (83%) and 19 parents (82%). Fifteen children (62,5%) and 19 parents (82%) found psychoeducation videos helpful or very helpful. Fourteen children (58%) and 14 parents (61%) found webcam sessions helpful or very helpful. Most negatively evaluated were the daily and weekly evaluation questions, rated as not helpful by 6 children (25%) and 3 parents (13%). Similarly, 6 children and 3 parents rated overall usefulness of the app as unhelpful. Reminders on the app was rated as unhelpful by four children and 3 parents (Fig. 2).

### Feasibility

No participants dropped out because of premature cessation. No participants dropped out because of premature cessation.No major deviations from the content of the eCBT treatment manual were reported. However, at the start of the eCBT program, several webcam sessions were cancelled by participants or ended in a phone call session due to initial hesitation on the part of patients or parents to use webcams, or technical problems on either the therapist or patient side. Unstable Internet connection was the most common technical problem reported in session integrity forms. Examples of other reasons were: one patient cancelled three scheduled webcam sessions because he had not downloaded the webcam application. Another came to see the therapist at the clinic in two instances when webcam sessions were scheduled. For two patients it was difficult to keep up webcam sessions (due to comorbid problems, i.e., ASD and eating disorder), resulting in only one completed session. This was compensated for by four face-to-face sessions in both cases.

**Fig. 3:**
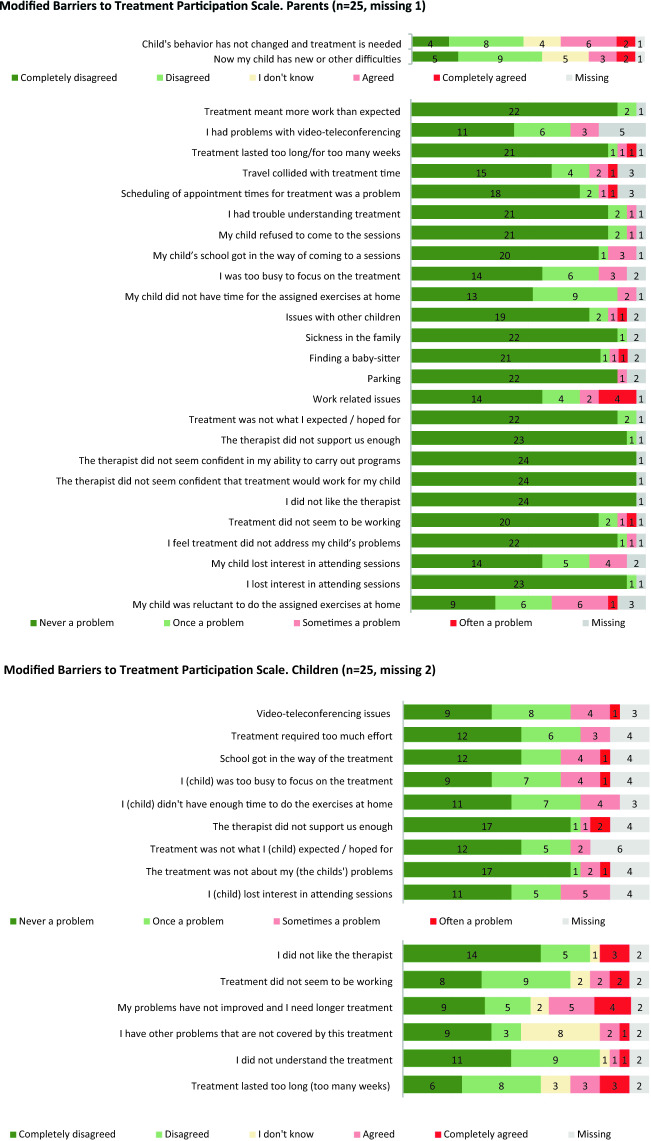
Modified Barriers to Treatment Participation Scale. Parents (n = 25, missing 1)

Results for the modified Barriers to Treatment Participation Scale (BTPS) showed that, parents listed 8 barriers and children listed 5 barriers to treatment as “often a problem” (see Fig. 3). The most frequent rated barrier to treatment was work related issues, endorsed by four parents. Two children endorsed, that the therapist did not support them enough.

### Treatment outcomes

Table 1 presents CY-BOCS, CGI-S and CGI-I measures at baseline, post-treatment, and at 3- and 6-month follow-up. There was a 63.8% mean symptom reduction on the CY-BOCS from baseline to post-treatment. At post-treatment evaluation, 19 patients scored below the clinical cut-off (CY-BOCS total score ≤ 15), and 15 patients met the criterion for clinical remission (CY-BOCS ≤ 10). Of the six patients with CY-BOCS scores above 15, three met the criterion for treatment response, showing at least 30% reduction of OCD severity. Of the 19 responders, 17 maintained their treatment gains with CY-BOCS scores further improving during 3-month and 6-month follow-ups.

## Discussion

This study explored feasibility, acceptability, and treatment outcomes of a newly developed enhanced cognitive behavioral therapy (eCBT) program for children and adolescents with OCD.

No patients dropped out of the eCBT study, suggesting that eCBT was a feasible treatment for children and parents. Interestingly, both parents and children reported very few barriers to treatment, and those reported were individually distributed single items. Only work-related issues were reported as “often a problem” by four parents (Fig. 3). Patient’s reluctance to do assigned homework was a common rated barrier to treatment. However, this is a well-known issue immanent to exposure therapy. The finding that nine children reported that they were not improved and need longer treatment might be explained by the three non-responders, the partial symptom reduction in three others, or by a temporary problem during difficult treatment phases*.*

Some technical issues were reported by both patients and therapists in the initial phase of the program, though these were relatively easily addressed (e.g., poor Internet connection). One patient’s OCD symptoms interfered with using the app. Initially they could not touch a cell phone due to fear of contamination, but after addressing this fear with the therapist they were able to engage in a full course of eCBT.

Overall, a smooth implementation of the program may have been facilitated because eCBT employs traditional CBT principles, which were well known to therapists. In addition, all therapists received a weekly supervision and assistance with technical problems from the eCBT project team. Moreover, the eCBT team expended a lot of effort in the developmental phase (i.e., consulting service users, exploring best possible technical solutions with IT experts) to make eCBT a user-friendly treatment for both patients and therapists. Finally, parents and children reported no major barriers that could preclude smooth implementation of eCBT.

To obtain detailed user feedback on acceptability, three questionnaires were used. The CSQ-8 tracks client satisfaction with the full eCBT package, while the TEQ evaluates the different parts of the package, and the UEQ reflects user experience with interactive products, specifically with the app. Overall, participants reported that they were highly satisfied with eCBT, which is reflected by high CSQ-8 mean total scores for both children and parents.

Evaluation of user experience overall demonstrated a neutral evaluation of all subscales of both parents and children (Fig. 1). High ranking of general satisfaction with eCBT package on the CSQ and the lower ranking of the user experience maybe because the hard exposure work was exclusively associated with the app. In addition, it is not surprising that children gave lower scores for app on UEQ novelty and attractiveness subscales. Children compared eCBT app to games, social media and other interactive apps, which are created for entertainment purpose and are usually more “fun” and more attractive for children.

In the evaluation of the different parts of the eCBT package (TEQ, Fig. 2), most children and parents evaluated the face-to-face as well as the videoconferencing sessions positively. One of the features of the app is to help structure and monitor exposure exercises. However, as in traditional CBT, motivation to engage in exposure is crucial. As expected, for the majority of children the app was an inspiring factor, motivating for more exposure, though others were more reluctant when it came to exposure, including use of the app. Regarding specific app features, children and parents were most positive about the psychoeducation videos. While most parents were positive about the assessment questions monitoring treatment progress, the aim of these questions seemed to be less clear for children. In general, parents’ evaluations of the app were more positive than children’s evaluations. A possible explanation for this might be the different roles of children and parents during treatment. Children may have been less positive about the app because of their experience with the “hard work” that exposure exercises entail, while parents’ experience of a gradual reduction of family accommodation and their role as supporters for their child may lead to more positive evaluations.

Overall, users appreciated both the face-to-face and the videoconferencing sessions. Webcam-based videoconferencing sessions offer not only the convenience of treatment at home but also the opportunity to practice therapist-guided ERP easily and realistically in the child’s natural environment, where obsessions are most often generated and compulsive behaviors performed. However, face-to-face sessions were rated positively by more participants than the videoconferencing sessions. This might in part reflect the fact that technical problems and a higher amount of uncertainty could have overshadowed the webcam sessions in the initial implementation phase. Our observation was that some children were easier to motivate and worked better in the context of face-to-face sessions, while for others exposure in natural situations at home with a webcam was more effective. Therefore, the opportunity to combine these treatment modalities to provide a more personalized approach, adapting the treatment schedule to individual needs and preferences, might further improve eCBT. This view is supported by a study of a 12-week clinician- and parent-supported Internet-based CBT program with low therapist intensity and with an average clinician time per patient per week of 17.5 min [15]. In this study, 46% of adolescents reported that they were satisfied with the Internet-delivered format, 50% were satisfied with the Internet format most of the time but would have liked to meet with a clinician occasionally, and 4% would have preferred face-to-face treatment.

Although the evaluation of the app was largely positive, the application of some features may need improvement. Participants mainly seemed to use those functions of the app which involved both therapist and patient. Functions like the toolbox, which children could explore and use on their own, were not used and may need to be better explained and integrated into the treatment. The same may apply to assessment questions monitoring treatment in the app. The most vital functions of the app were the OCD symptom inventory and the list for ERP exercises, including monitoring of the accomplishment of exercises. These features allowed a continuous communication between patient and therapist and contributed to improving both session structure and communication.

As is the case with most apps, most users do not utilize all possible functions. To concentrate on the core functions in daily use, with the possibility of applying more sophisticated features when needed, may be a good strategy for future apps [15]. On the other hand, to keep an application simple and straightforward might improve both the attractiveness of the tool and the compliance of users. Reminders for exercises were an ambiguous tool; children with high motivation for treatment did not need reminders, while those with little motivation could experience reminders as annoying.

In general, there was a large improvement in OCD symptoms, with 63.8% mean reduction of CY-BOCS total scores from baseline to post-treatment. Nineteen out of 25 patients had OCD symptoms below the clinical cut-off and 15 of them fulfilled the criterion for remission after eCBT. Three patients responded to treatment, with a large reduction of CY-BOCS total scores, although at post-treatment their CY-BOCS scores were still above the clinical cut-off. One patient showed no treatment response at all. This patient was first treated at an inpatient unit for anorexia nervosa and subsequently referred for OCD treatment. Her engagement in eCBT was limited: she had little motivation or energy to perform ERP exercises. Later, as her anorexia nervosa symptoms improved, she was able to apply principles learned during eCBT. This patient started exposure exercises on her own and subsequently was deemed to be a responder at 6-month follow-up with a CY-BOCS total score of 5. The two other non-responders had only minor reductions of CY-BOCS scores.

While a direct comparison should not be drawn between large interventional studies and this exploratory study, we noticed similar trends between eCBT and the Nordic long-term OCD treatment study (NordLOTS), as eCBT employs key elements of the NordLOTS manual. In NordLOTS (the largest study to date of the effects of CBT for pediatric OCD), 72.6% of the participants were responders (CY-BOCS total score ≤ 15), and mean reduction on the CY-BOCS was 52.9% (SD 30.9) at post-treatment [31]. Our results are also in line with other studies using Internet technology to deliver CBT. Storch et al. [36], for example, reported a 56.1% reduction of CY-BOCS total score after 14 sessions of webcam-delivered CBT. Farrell et al. [37] reported a 49% CY-BOCS score reduction after 3 face-to-face CBT sessions, followed by maintenance sessions via webcam. Other studies applying various degrees of Internet technology to deliver CBT to children with OCD reported somewhat lower reductions of CY-BOCS scores after treatment [18].

This study has several limitations and should be viewed in its methodological context. The findings are based on a relatively small number of participants, limiting the generalizability of the current findings. Another limitation is the relatively large difference in the distribution of face-to-face versus webcam sessions between patients and the number of therapists (8) who treated 25 patients. This may have contributed to a considerable variability of our data. The app was developed for the Android mobile system only, and children who had an iPhone had to borrow an Android phone from the study. Strengths of this study included the fact that assessments were based on reliable tools and a multi-perspective approach, taking the views of children, parents, and therapists into account, and that CY-BOCS evaluations were performed by an independent rater and not the therapists. In addition, another strength is the follow-up assessment at 6 months. The sample included patients with moderate to severe OCD and high rates of comorbid disorders, which seemed to be representative of the patient population usually seen in our specialized OCD treatment unit.

## Conclusions

In this study, eCBT for pediatric OCD was a feasible and acceptable intervention demonstrating positive treatment outcomes. Opportunities to combine face-to-face and webcam treatment modalities as part of a more personalized approach, adapting the treatment schedule to individual needs and preferences, might further improve eCBT.

## Data Availability

The data generated or analysed during this study are included in this published article [and its supplementary information files] and are also available from the corresponding author on reasonable request.

## Abbreviations

ADHD: Attention deficit hyperactivity disorder
ASD: Autism spectrum disorder
BTPS: Barriers to Treatment Participation Scale
CBT: Cognitive behavioral therapy
CGAS: Children’s Global Assessment Scale
CGI: Clinical Global Impression Scale
CSQ-8: The Client Satisfaction Questionnaire-8
CY-BOCS: Children’s Yale-Brown Obsessive–Compulsive Scale
DSM: The Diagnostic and Statistical Manual of Mental Disorders
eCBT: Enhanced cognitive behavioral therapy
ERP: Exposure and response prevention
GCP: Good Clinical Practice
iCBT: Internet cognitive behavioral therapy
ISRCTN: International Standard Randomised Controlled Trial Number
IT: Information Technology
K-SADS-PL: The Schedule for Affective Disorders and Schizophrenia for School-Age Children –Present and Lifetime Version
NordLOTS: The Nordic long-term OCD treatment study
OCD: Obsessive Compulsive Disorder
TEQ: Treatment Evaluation Questionnaire
UEQ: User Experience Questionnaire
RCT: Randomized controlled trial
